# Whole Genomic Sequence and Replication Kinetics of a New Enterovirus C96 Isolated from Guangdong, China with a Different Cell Tropism

**DOI:** 10.1371/journal.pone.0086877

**Published:** 2014-01-30

**Authors:** Jing Lu, Huanying Zheng, Yong Zhang, Xue Guo, De Wu, Hui Li, Leng Liu, Hanri Zeng, Lina Yi, Ling Fang, Yanling Mo, Wenbo Xu, Changwen Ke

**Affiliations:** 1 Guangdong Provincial Center for Disease Control and Prevention, Guangzhou, China; 2 Guangdong Provincial Institution of Public Health, Guangdong Provincial Center for Disease Control and Prevention, Guangzhou, China; 3 WHO WPRO Regional Polio Reference Laboratory and Ministry of Health Key Laboratory for Medical Virology, National Institute for Viral Disease Control and Prevention, Chinese Center for Disease Control and Prevention, Beijing, China; Centers for Disease Control and Prevention, United States of America

## Abstract

Enterovirus 96 (EV-C96) is a newly described serotype within the *enterovirus C* (*EV-C*) species, and its biological and pathological characters are largely unknown. In this study, we sequenced the whole genome of a novel EV-C96 strain that was isolated in 2011 from a patient with acute flaccid paralysis (AFP) in Guangdong province, China and characterized the properties of its infection. Sequence analysis revealed the close relationship between the EV-C96 strains isolated from the Guangdong and Shandong provinces of China, and suggested that recombination events occurred both between these EV-C96 strains and with other EV-C viruses. Moreover, the virus replication kinetics showed EV-C96 Guangdong strain replicated at a high rate in RD cells and presented a different cell tropism to other strains isolated from Shandong recently. These findings gave further insight into the evolutionary processes and extensive biodiversity of EV-C96.

## Introduction

Enteroviruses (family *Picornaviridae*, order *Picornavirales*) are small RNA viruses associated with several human diseases ranging from asymptomatic or mild infections to more severe diseases such as hand, foot, and mouth disease [1], [2], meningitis [3], encephalitis [4], pericarditis [5], and acute flaccid paralysis [6]. All enteroviruses contain a genome of approximately 7,500 bases and are known to have a high mutation rate due to low-fidelity replication and frequent recombination [7], [8], [9]. Recently, VP1 region sequencing has supplanted neutralization testing as the standard for enterovirus typing [10]. Based on their molecular properties, human enteroviruses are currently grouped into four species: *enterovirus A* (*EV-A*), *EV-B*, *EV-C*, and *EV-D* [11]. Molecular typing of serologically non-typable isolates has led to the discovery of a large number of new enterovirus types [12], [13], [14], [15].

Enterovirus 96 (EV-C96) is a recently described genotype in the species *EV-C* [8]. The prototype virus was isolated in 2000 from a stool specimen of a patient with acute flaccid paralysis (AFP) in Bangladesh [16]. Thereafter, EV-C96 strains have been reported in Cambodia [17], Slovakia [8], Finland [8], [18], Bolivia [19], the Philippines [20], and China [21], [22], [23], suggesting that EV-C96 might be a widespread virus. Although the sequences of several EV-C96 genomes have been reported, the biological and pathogenic properties of this virus are still poorly defined.

In this study, we reported an EV-C96 strain isolated in December 2011 from a patient with AFP in Guangdong province, China. Analysis of the complete genome sequence revealed intertypic and intratypic recombination associated with virus evolution. The replication kinetics of EV-C96 was determined in different types of cells, and EV-C96 Guangdong strain displayed a different cell tropism to strains recently isolated from Shandong province, China.

## Results

### Viral isolation and serotyping

The virus was successfully isolated from RD cells on the sixth day of cultivation. In contrast, no significant CPE was observed in other three cell lines used (HEp-2, Vero, and L20B) after 14 days. The virus serotype could not be determined by a neutralization test using enterovirus antiserum pools. Therefore, the virus was initially characterized via molecular methods, by partial sequencing of the capsid protein *VP1* coding region and BLAST analysis, and it was identified as EV-C96.

### All EV-C96 stains form three clusters

**Figure 1:**
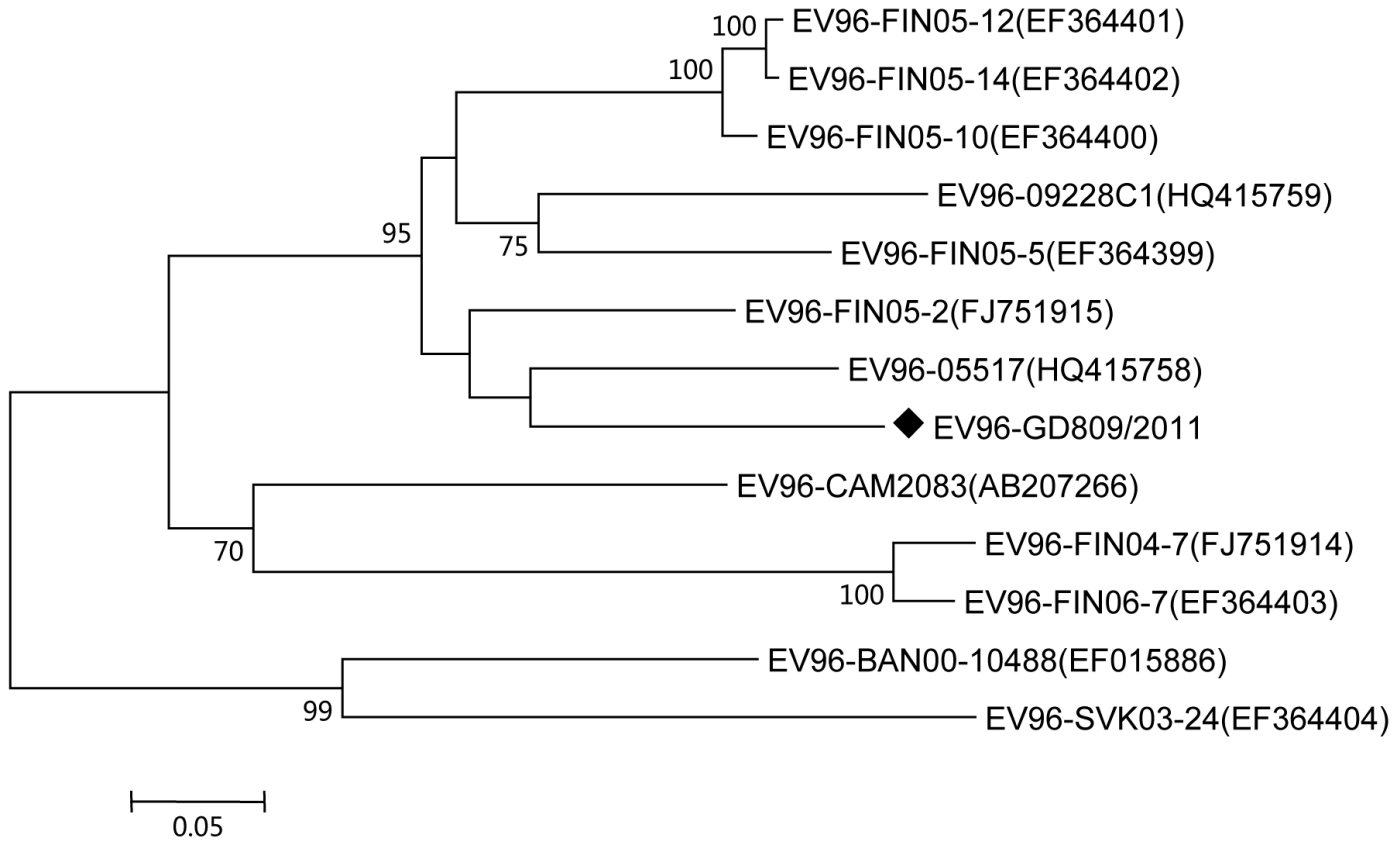
Phylogenetic tree of the VP1 coding region of the EV-C96 strains available in GenBank. The locations of the Guangdong strains are indicated by a black diamond. Bar, nucleotide distance as substitutions per site. Only bootstrap values of over 70% are shown.

The genome length of GD809/2011 was 7,469 nt, which was predicted to encode 2,218 amino acids of polypeptides. The coding sequences were flanked by a 5′ untranslated region (5′-UTR) of 745 nt, and a 3′-UTR of 68 nt. The overall base composition of the genome was 30.1% A, 22.9% G, 22.6% C, and 24.4% U. The phylogenetic tree based on the complete *VP1* sequence suggested that all the reported EV-C96 strains could be grouped into three clusters with more than 70% bootstrap support (Fig. 1). GD809/2011 was more closely related to other EV-C96 strains from China as well as strains from Finland (isolated in 2005) and grouped with them to form one cluster. Other strains isolated from Finland (isolated in 2004 and 2006) and Cambodia formed the second cluster, and strains from Bangladesh and Slovakia formed the third cluster.

### GD809/2011 showed intertypic and intratypic recombination

**Figure 2:**
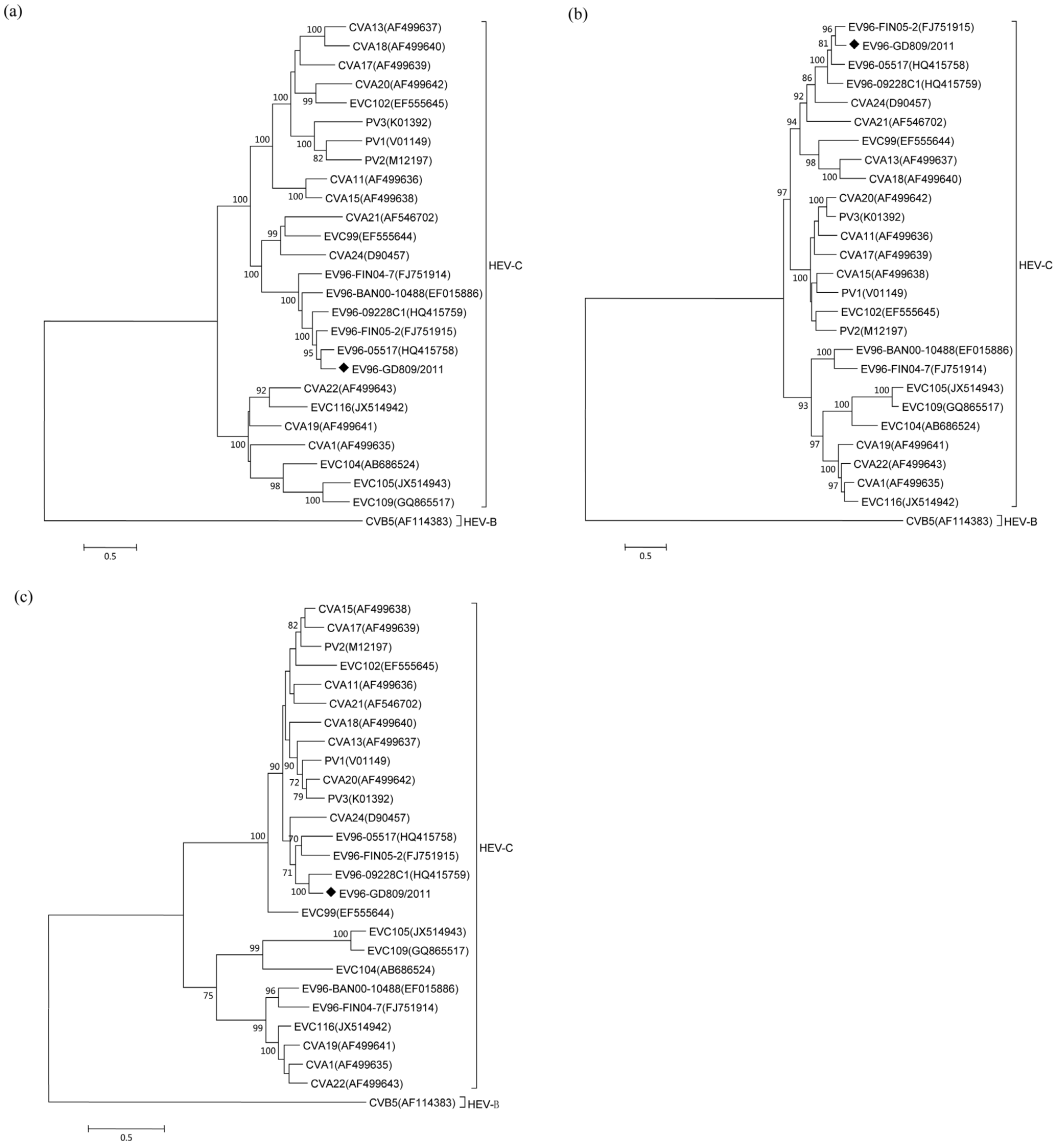
Phylogenetic trees constructed based on the *P1* (a), *P2* (b), and *P3* (c) coding regions of EV-C strains. The five EV-96 strains with available full genome sequences were included. The locations of the Guangdong strains are indicated by a black diamond. Bar, nucleotide distance as substitutions per site. Only bootstrap values of over 70% are shown.

**Figure 3:**
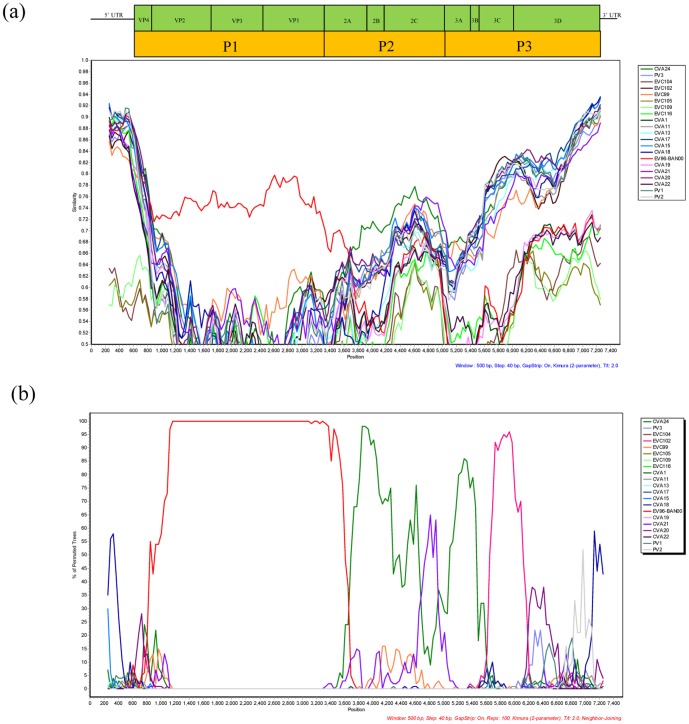
Similarity plot (a) and bootscanning analysis (b) of the complete EV-C genomes using a sliding window of 500 nt moving in 40-nt steps. The EV-96 strain GD809/2011 was used as a query sequence.

Genomic recombination is known to contribute to the genetic variation and evolution of enteroviruses [24]. Similarity plots and bootscanning analyses were next used to detect recombination events in GD809/2011. The phylogenetic analysis based on the *P1* region indicated that all EV-C96 strains formed a monophyletic group that was separated from CVB5 as the outgroup (Fig. 2a). However, for the *P2* and *P3* regions, the EV-C96 isolates were not monophyletic. In the *P3* region, the EV-C96 strains shared 70%–86% similarity with each other and 68%–84% similarity with other EV-C strains (Fig. 2c). Similar results were also obtained by analyzing the *P2* region (Fig. 2b). This incongruity between the phylogenetic trees based on the *P1*, and those based on the *P2* and *P3* regions suggested intertypic recombination likely occurred between GD809/2011 and other species of EV-C. Therefore, similarity plots and bootscanning analyses were further performed to identify the regions of recombination in the EV-C96 isolates. The complete genome sequence of GD809/2011 was used as the query and was compared with the EV-C96 prototype strain (BAN00-10488) and other EV-C prototype strains. As expected, higher sequence similarity between GD809/2011 and the EV-C96 prototype strain was observed in the structural *P1* region with nearly 100% bootstrap support. Curiously, in the *P2* and *P3* coding regions, GD809/2011 was more closely related to CVA24 and EV-C102 with greater than 90% bootstrap values (Fig. 3a&b). The calculated first breakpoint was situated at the 3′-terminus of the 2A region in the nucleotide 3650, and the second breakpoint was located at the 3′-terminus of the 3C region in the nucleotide 5610.

**Figure 4:**
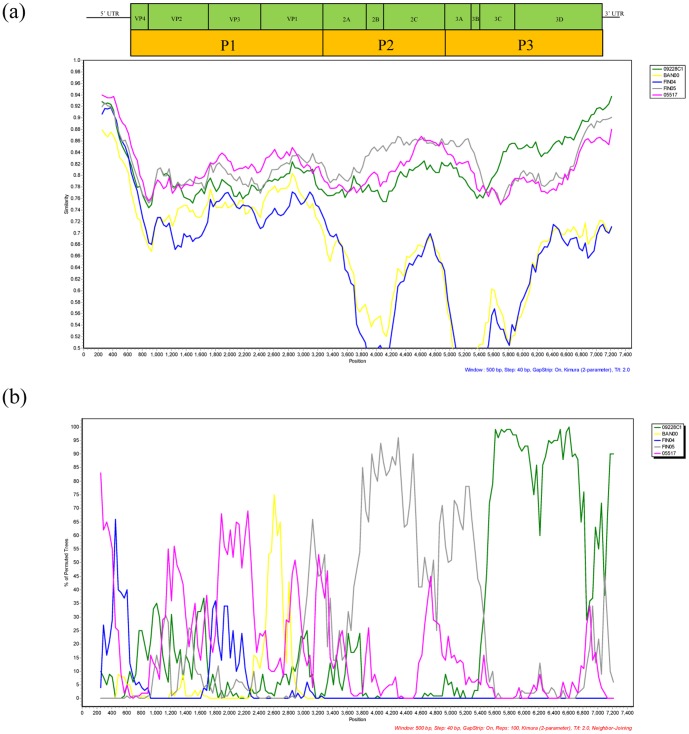
Similarity plot (a) and bootscanning analysis (b) of the complete genome of the newly isolated EV-C96 strain GD809/2011 with other EV-C96 strains. Analysis was performed by using a sliding window of 500-nt steps. GD809/2011 was used as a query sequence.

Moreover, the phylogenetic analysis also suggested that intratypic recombination may occur among EV-C96 strains. In the *P1* and *P2* regions, the EV-C96 strains GD809/2011, 05517, and FIN05-2 were grouped together and were separated from 09228C1 (Fig. 2a & 2b). In contrast, in the *P3* region, GD809/2011 was more similar to 09228C1 than to 05517 and FIN05-2 (Fig. 2c). The similarity plots and bootscanning graphs also demonstrated that strain 09228C1 was less similar to GD809/2011 in the *P1* region, but more similar in the *P3* region (Fig. 4a&b). The recombinant region mainly located in the *3C* coding region, a hot spot for recombination in other enterovirus serotypes [25].

### Analysis of virus replication in RD and HEp-2 cells

**Figure 5:**
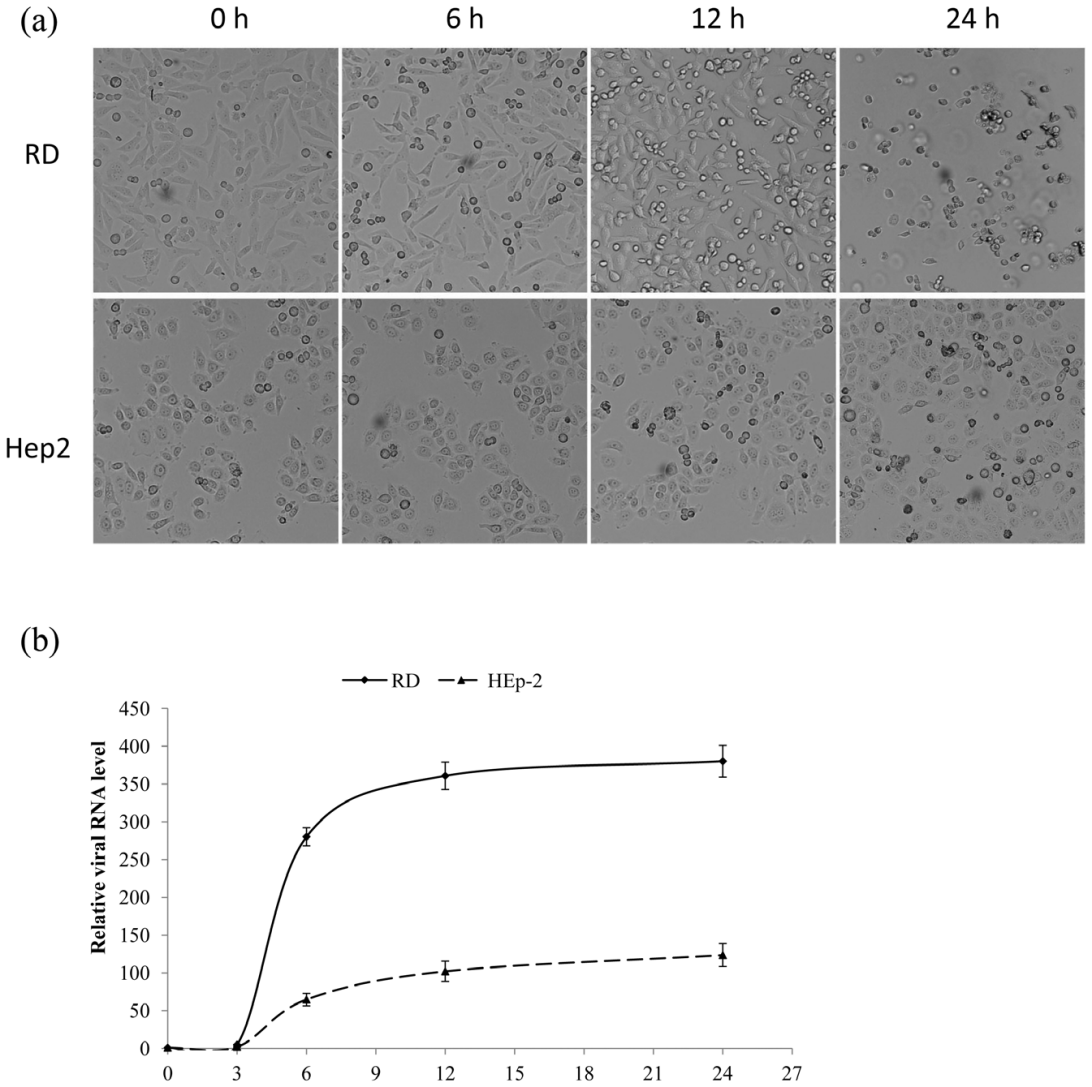
Cytopathic effects (CPEs) and virus replication kinetics of GD809/2011 infection. RD and Hep-2 cells were infected with GD809/2011 at an MOI of 1. (a). The CPEs were shown by the cell morphological changes at 0, 6, 12, and 24 h p.i. (original magnification, ×100); (b). Intracellular viral RNA levels were measured at 0, 3, 6, 12, and 24 h p.i. by qRT- PCR. The relative viral RNA level at 0 h p.i. was set as 1, and the viral RNA levels at other time points were expressed as fold changes. Data shown are the mean ± SD of three independent experiments.

The most recently isolated EV-C96 Shandong strains, 05517 and 09228C1, only induce CPE in HEp-2 cells but not in RD, Vero or L20B cells [21]. In contrast, GD809/2011 showed a different cell tropism which induced significant CPE in RD cells but not in other types of cells during viral isolation. To confirm the cell tropism and better understand the biological characteristics of GD809/2011, we analyzed the virus replication kinetics in both RD and HEp-2 cells. In RD cells, GD809/2011 infection induced CPE as early as 6 h p.i., and infected cells underwent significant death and detached from the surface of culture dishes at 12 h p.i. (Fig. 5a). At 24 h p.i., more than 80% of the cells were detached from the surface. In contrast, no significant CPE was observed in HEp-2 cells until 12 h p.i.

The virus replication kinetics were calculated by measuring the cellular viral RNA levels at different time points p.i. In RD cells, intracellular viral RNA levels began to increase as early as 3 h p.i., and the exponential phase was from 3 to 6 h p.i. The viral RNA level at 6 h p.i. was 280 times higher than the level at 0 h p.i. (Fig. 5b). The virus replication rate in HEp-2 cells was significantly lower than in RD cells, and most HEp-2 cells were still healthy at 24 h p.i. (Fig. 5).

## Discussion

Recently, EV-C96 was recognized as a new member of EV-C species following the isolation of strains in Asia, South America and Europe [8], [18], [19], [23]. The virus has been also isolated from patients with AFP and healthy individuals in Yunnan and Shandong provinces, demonstrating the circulation of EV-C96 in China [21], [23]. However, little is known about the biological and pathological properties of EV-C96 infection.

Extensive studies have shown that intertypic and intratypic recombination events are common among EV-A, EV-B, and EV-C strains [26], [27], [28], and EV-C96 is no exception. Previous studies suggested that potential recombination events occurred between EV-C96 and CVA1, CVA19, CVA22, and PV1 [8], [29]. In this study, intertypic recombination was observed in the *P2* and *P3* regions of GD809/2011, and the donor sequence was derived from another EV-C (probably CVA24 or EV-C102). This could be the case, although both CVA24 and EV-C102 are rarely detected. Considering the high rate of asymptomatic enterovirus infections, the prevalence of these Human enteroviruses (HEVs) may be underestimated. Moreover, co-infection of EV-C96 and CVA24 was observed in a patient with AFP in Slovakia early in 2003 [8]. No intratypic recombination events were previously reported due to the lack of complete EV-C96 genome sequences data. In this study, potential intratypic recombination between GD809/2011 and 09228C1 was revealed by phylogenetic tree analyses based on different regions of the viral genome. The recombination events that occurred between the Guangdong strain and Shandong stain in the *P3* region of the genome suggested that these two strains may have been co-circulating in mainland China for a long time.

Studies of other enteroviruses indicate that HEVs evolve quickly due to the high frequency of errors in RNA synthesis (roughly 10^−4^ per base per replication cycle) and frequent recombination [24], [30], [31], [32]. The predominance of mutations and recombination events may have resulted from the rate of viral replication and translation, the ability of the virus to spread, and the capability of the virus to induce cell death. Therefore, sequence variance and recombination may play a role in the evolution of HEV strains by improving the overall fitness of the virus. In this study, we observed that GD809/2011 have a different cell tropism from the other strains isolated from Shandong, despite relative high sequence identity (Fig. 1). In RD cells, GD809/2011 underwent fast replication from 3 to 6 h p.i., and intracellular viral RNA was rapidly accumulated (Fig. 5b). The intracellular viral RNA level was increased by more than 56-fold within this time period. Little increase was observed after 12 h p.i., which suggested that the cells may not sustain virus replication and underwent apoptosis (Fig. 5a). Similar results were also reported for poliovirus and EV-A71 infection [33]. In contrast, GD809/2011 replicated more slowly in HEp-2 cells, and it induced significant cell death only at 24 h p.i. (Fig. 5a&b). The different cell tropisms observed between the Guangdong and Shandong EV-C96 strains may be due to sequence variance caused by mutation or recombination. Studies of other enteroviruses suggested that sequence variations in *UTRs*, the *VP1* capsid region, and the *3D* polymerase could affect virus infection and replication capability *in vitro* and *in vivo* [30], [34], [35]. Therefore, recombinations may benefit virus adaptation to new or changing environments by providing large genome segments that may be pre-adapted to new host cells. In future study, more EV-C96 virus strains and genome sequences need to be included to identify the sequences associated with the virulence.

In conclusion, we reported the complete genome sequence of an EV-C96 strain (GD809/2011) isolated in Guangdong province, China. The complete genome was characterized, and its phylogenetic relationships with other EV-C96 strains were analyzed. Potential intertypic and intratypic recombination was observed in non-structural regions. The virus replication kinetics in RD and HEp-2 cells revealed that this newly isolated virus had a different cell tropism than other strains isolated from Shandong, China. As viral kinetics are important for demonstrating viral activities in host cells, the present study provided not only the complete genome sequence of GD809/2011, but also basic information on the viral-host interactions and pathogenesis of this virus.

## Methods

### Ethics Statement

The study was approved by the institutional ethics committee of Center for Disease Control and Prevention of Guangdong Province (Guangdong CDC). This study did not involve human participants or human experimentation, and the only human materials used were stool samples collected from AFP patients at the instigation of the Ministry of Health P. R. of China for public health purposes. The written consent was early prepared and signed by the patients or their guardian(s) when samples collected.

### Sample collection

Guangdong EV-C96 strain (strain AFP809/GD/CHN/2011, hereafter referred to GD809/2011) was isolated in 2011 from a 26-month-old girl from Zhanjiang City who developed AFP. The patient's illness started with a cough, runny nose, and a feeling of being tired 15 days before admission. Sudden weakness in the limbs, excessive drowsiness, and occasional muscle twitching were observed on the day of the admission.

For research purposes, a stool sample was collected from the patient at hospital admission with informed consent from her parents and transported to the laboratory in a refrigerated package and then processed according to standard protocols recommended by the World Health Organization (WHO). Human rhabdomyosarcoma (RD) cells (CCL-136, ATCC, USA), human laryngeal epidermoid carcinoma (HEp-2) cells (CCL-23, ATCC), African green monkey kidney (Vero) cells (CCL-81, ATCC), and recombinant mouse L cells expressing the human poliovirus receptor (L20B) (CCL-96, ATCC) were used to isolate viruses from the stool sample. The inoculated cells were examined under microscopy daily for 14 days for the appearance of cytopathic effect (CPE). The wells in which CPE was observed were recorded, and then the cultures were collected for neutralization testing and molecular characterization. Micro neutralization assays were carried out in 96-well tissue culture plates using EV antiserum pools (National Institute for Public Health and the Environment, RIVM, the Netherlands).

### Nucleic acid extraction and virus identification

For molecular typing, nucleic acid was extracted from the collected culture using the QIAamp Viral RNA mini kit (QIAGEN, USA) according to the manufacturer's instructions. Viral RNA was eluted by adding 60 µL of sterile, nuclease-free water and was stored at −80°C until use. RT-PCR was performed according to the method developed by Nix *et al.* [36]. The PCR products were purified using the QIAquick PCR purification kit (QIAGEN), and then used for nucleotide sequencing. The sequences were analyzed with the Basic Local Alignment Search Tool (BLAST) server at the National Center for Biotechnology Information (NCBI) and the EV serotype was determined according to a previously described molecular typing method [37]. The primers used to amplify the complete genome were designed by ‘primer-walking’ and are available upon request. SuperScript III One-Step RT-PCR Platinum Taq (Invitrogen, 12574-035) was used for the amplification. The PCR products were gel purified and then sequenced twice in both directions using the same forward and reverse primers as those used in the PCR. The complete genome sequence of GD809/2011 was deposited in the GenBank database (accession number: KF495604).

### Analysis of virus replication kinetics

The titers of isolated virus were measured using CPE microtitration assays and were expressed as the 50% tissue culture infective dose (TCID_50_) per mL according to the Kärber formula [38].

The virus replication kinetics was analyzed in RD and HEp-2 cells. Briefly, the cells were plated in 12-well plates one day before infection. Just prior to infection, the cells were washed twice with PBS and mock infected or infected with GD809/2011 EV-C96 at a MOI of 1. Time was set to zero after 1 h of adsorption. The supernatant was removed, and the cells were washed twice with PBS to remove unattached virus before adding 1 mL of DMEM medium containing 10% FBS to each well. The cells were then cultured at 37°C in 5% CO_2_. Cell morphology was monitored using a phase-contrast microscope, and the infected cells were harvested at different time points to isolate RNA. Quantitative real-time PCR (qRT-PCR) was performed as previously described [39] using the ABI 7500 Real-Time PCR system (Applied Biosystems, USA), the QuantiTect SYBR Green PCR Kit (Qiagen), and specific forward EV-C96-VP1F (5′-ACCTTTGTGTTGACTGAGAGATAC-3′) and reverse EV-C96-VP1R (5′-GCAAACCCGTCGTAGAAATGG-3′) primers targeting the VP1 region. GAPDH was used as an internal control. The relative viral RNA level at 0 h post infection (p.i.) was set to 1. The viral RNA level at other time points was expressed as fold the change compared with the viral level at 0 h p.i. Each experiment was performed in triplicate, and the data are reported as the mean ± standard deviation (SD).

### Genome analyses

The sequence of the newly isolated virus was compared with those of other previously reported EV-C96 strains and a phylogenetic tree was constructed in a maximum likelihood (ML) framework using MEGA version 5.1 with the general time reversible GTR+G model and 500 replicates [40], [41]. Potential intertypic and intratypic recombination was analyzed using the SimPlot 3.5.1 program with a 500-nt window moving in 40-nt steps and Jukes-Cantor correction [42]. The sequences used for comparison included the previously reported EV-C96 strains BAN00-10488 (EF015886), FIN04-7 (FJ751914), FIN05-2 (FJ751915), FIN06-7A (EF364403), FIN05-14 (EF364402), FIN05-12 (EF364401), FIN05-10 (EF364400), FIN-05-5 (EF364399), FIN06-9 (EU481511), and SVK03-24 (EF364404) as well as other EV-C viruses CVA1 (AF499635), CVA11 (AF499636), CVA13 (AF499637), CVA17 (AF499639), CVA19 (AF499641), CVA20 (AF499642), CVA21 (AF546702), CVA22 (AF499643), CVA24 (D90457), EV-C99 (EF555644), EV-C102 (EF555645), EVC104 (AB686524), EV-C105 (JX514943), EV-C109 (GQ865517), EV-C116 (JX514942), poliovirus type 1 (V01148), poliovirus type 2 (X00595), and poliovirus type 3 (K01392).
